# The Dependence of Hydrophobic Interactions on the Shape of Solute Surface

**DOI:** 10.3390/molecules29112601

**Published:** 2024-06-01

**Authors:** Yu-Zhen Liu, Yan-Nan Chen, Qiang Sun

**Affiliations:** Key Laboratory of Orogenic Belts and Crustal Evolution, Ministry of Education, The School of Earth and Space Sciences, Peking University, Beijing 100871, China; liuyuzhen@stu.pku.edu.cn (Y.-Z.L.); 2301210129@stu.pku.edu.cn (Y.-N.C.)

**Keywords:** water, hydrogen bonding, hydrophobic interactions, solute shape

## Abstract

According to our recent studies on hydrophobicity, this work is aimed at understanding the dependence of hydrophobic interactions on the shape of a solute’s surface. It has been observed that dissolved solutes primarily affect the structure of interfacial water, which refers to the top layer of water at the interface between the solute and water. As solutes aggregate in a solution, hydrophobic interactions become closely related to the transition of water molecules from the interfacial region to the bulk water. It is inferred that hydrophobic interactions may depend on the shape of the solute surface. To enhance the strength of hydrophobic interactions, the solutes tend to aggregate, thereby minimizing their surface area-to-volume ratio. This also suggests that hydrophobic interactions may exhibit directional characteristics. Moreover, this phenomenon can be supported by calculated potential mean forces (PMFs) using molecular dynamics (MD) simulations, where different surfaces, such as convex, flat, or concave, are associated with a sphere. Furthermore, this concept can be extended to comprehend the molecular packing parameter, commonly utilized in studying the self-assembly behavior of amphiphilic molecules in aqueous solutions.

## 1. Introduction

The term “hydrophobic” stems from the Greek words “hydros”, meaning water, and “phobos”, signifying fear. Hydrophobic effects denote the inclination of nonpolar molecules or segments thereof to cluster in aqueous environments (or to be expelled from water, forming specific complexes in solutions). They are widespread and are considered the primary driving force behind numerous biological and biochemical phenomena, including the spontaneous organization of molecules with both hydrophilic and hydrophobic regions in water [1,2,3]. It is well-established that dissolved molecules aggregate to form structures with diverse geometric configurations, such as spherical, globular, rod-like, or bilayers [1,2,3]. As a result, self-assembly processes are increasingly exploited to design and produce highly organized materials with functional or physical properties distinct from those of their individual components. Therefore, hydrophobic effects have attracted considerable attention, and numerous works [4,5,6,7,8,9,10,11,12,13,14,15,16,17,18] have been carried out.

In the early 1940s, Frank and Evans [4] introduced the renowned “iceberg” model of hydrophobic effects, suggesting that water molecules assemble into an ordered “cage” structure around small hydrophobic solutes like argon or methane. However, dissolving large solutes in water can disrupt the hydrogen bonds of water molecules [5], leading to an enthalpic penalty. To explore the relationship between hydrophobic interactions and solute size, Lum, Chandler, and Weeks (LCW) [6,7,8,9] developed a theoretical framework. In the LCW theory, Gaussian density fluctuations for small solutes and the physics of interface formation for large solutes reflect the dependence of hydrophobic hydration on solute size. Additionally, a crossover between small and large solutes is anticipated at the nanometer length scale [8]. In our recent works [16,17,18], we derived hydration free energy to investigate the mechanism of hydrophobic effects. As solute size increases, it is reasonably partitioned into initial and hydrophobic solvation processes [16,18]. Moreover, distinct behaviors of solutes in water can be anticipated during the initial and hydrophobic solvation processes, leading to dispersed or aggregated distributions in solutions [16,17,18].

Mathematically, solute curvature can broadly be categorized as positive curvature (convex), zero curvature (flat surface), and negative curvature (concave). Experimental and computational studies [19,20] have revealed differences in the solvation of convex, flat, and concave surfaces. This differentiation is ascribed to the influence of external surfaces on water hydrogen bonding. On a concave surface, water molecules are unable to form as many connections with other water molecules as they can on more exposed surfaces, such as flat and convex surfaces [19,20].

This study is aimed at investigating the dependence of hydrophobic interactions on the geometric shape of the solute surface. Owing to hydrophobic interactions, the solutes tend to be aggregated in a specific direction to minimize the ratio of the surface area to the volume of solutes [16,18]. Therefore, hydrophobic interactions may be closely related to the shape of the solute surface. This relationship can be elucidated through the PMF calculations obtained via MD simulations, where a sphere is associated with various surfaces, including convex, flat, or concave. Moreover, this can be extended to elucidate the molecular packing parameter, a metric commonly employed to investigate the self-assembly dynamics of amphiphilic molecules in aqueous solutions.

## 2. Results and Discussion

### 2.1. Thermodynamic Analysis

In thermodynamics, Gibbs free energy (ΔG) serves as a useful tool for determining whether a process is favored (ΔG < 0) or disfavored (ΔG > 0). When solutes are dissolved in water, the total thermodynamic functions can be expressed as follows,

(1) $${\Delta G}_{\mathrm{Total}}={\Delta G}_{\mathrm{Solute}–\mathrm{solute}}+{\Delta G}_{\mathrm{Solute}–\mathrm{water}}+{\Delta G}_{\mathrm{Water}–\mathrm{water}}$$

Before the solutes are affected by the direct solute–solute interactions, they must approach each other in solution. This process is undoubtedly related to the changes in ΔG associated with water–water (ΔG_Water–water_) and solute–water (ΔG_Solute–water_) interactions. To understand the mechanisms underlying hydrophobic effects, it is essential to investigate the structure of liquid water, and the effects of solutes on water structure.

OH vibrations are sensitive to the hydrogen bonding network of water, and are widely used to investigate the structure of liquid water [21,22]. Water molecular clusters, denoted as (H_2_O)_n_, offer insights into the relationship between OH vibrations and hydrogen-bonded networks. Based on our recent studies [23], when the three-dimensional hydrogen bonds appear (≥6), distinct OH vibrational frequencies correspond to various hydrogen-bonded networks within the first shell of a water molecule (local hydrogen bonding), while the effects of hydrogen bonding beyond the first shell on OH vibrations are weak.

To understand the structure of water, various structural models have been proposed, and these can be roughly categorized into two groups: mixture and continuum models [24,25,26,27,28,29,30,31]. In Raman spectroscopic studies [23] on liquid water, the local statistical model (LSM) has been proposed. This model suggests that a water molecule interacts with neighboring water molecules (in the first shell) through various local hydrogen-bonded networks, such as DDAA (double donor-double acceptor, tetrahedral hydrogen bonding), DDA (double donor-single acceptor), DAA (single donor-double acceptor), DA (single donor-single acceptor), and free OH vibrations [23].

As a solute is embedded into water, a solute–water interface is expected to appear, which may affect the structure of water. When the three-dimensional hydrogen bonds occur in water, the OH vibrations may be closely related to the local hydrogen bonds of a water molecule [23]. Consequently, the solute predominantly affects the topmost water layer at the solute–water interface, referred to as interfacial water. Hence, during the time in which a solute is dissolved in water, it can be divided into interfacial and bulk water. Therefore, the effects of the solute on the water structure (ΔG_Solute–water_) may be closely associated with the solute–water interface.

Based on a vibrational sum frequency generation (SFG) study [32] on the air–water interface, no tetrahedral (DDAA) hydrogen bonding is expected to be present in the interfacial water. Once the ratio of the interfacial water layer to volume is determined, it is used to determine the Gibbs free energy between the solute and water (ΔG_Solute–water_).

(2) $${\Delta G}_{\mathrm{Solute}–\mathrm{water}}={\Delta G}_{\mathrm{DDAA}}\cdot R_{\mathrm{Interfacial}\;\mathrm{water}/\mathrm{volume}}\cdot n_{\mathrm{HB}}$$

Here, ∆G_DDAA_ represents the Gibbs free energy of DDAA (tetrahedral) hydrogen bonding, R_Interfacial water/volume_ denotes the molecular number ratio of the interfacial water layer to volume, and n_HB_ represents the average number of hydrogen bonds per molecule. At 293 K and 0.1 MPa, ∆G_DDAA_ is calculated to be −2.66 kJ/mol.

**Figure 1:**
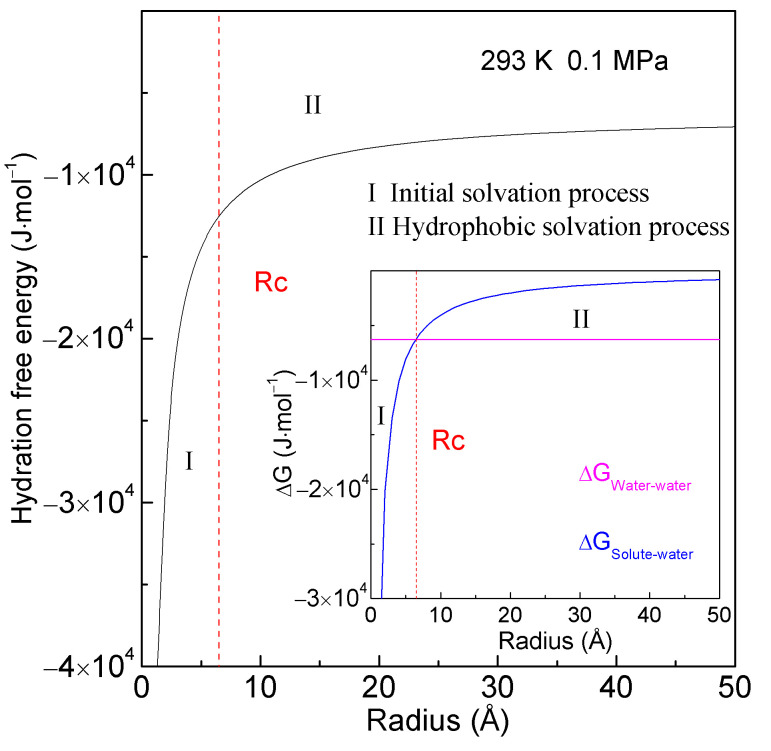
Hydration free energy at 293 K and 0.1 MPa. It is the sum of the Gibbs energies of the bulk (∆G_Water–water_) and interfacial (∆G_Solute–water_) water. With reference to Rc, it is divided into initial and hydrophobic solvation processes, respectively.

The hydration free energy represents the change in Gibbs free energy as an ion or molecule is transferred from a vacuum or gas phase to a solvent. For a spherical solute, the ratio of interfacial water layer to volume (R_Interfacial water/volume_) can be expressed as 4∙r_H2O_/R, where R is the radius of the solute [16]. Consequently, when the spherical solute is immersed in water, the hydration free energy can be expressed as follows (Figure 1):

(3) $${\Delta G}_{\mathrm{Hydration}}={\Delta G}_{\mathrm{Water}–\mathrm{water}}+{\Delta G}_{\mathrm{Solute}–\mathrm{water}}={\Delta G}_{\mathrm{Water}–\mathrm{water}}+\frac{8\cdot {\Delta G}_{\mathrm{DDAA}}\cdot r_{H2O}}{R}$$

where ∆G_Water–water_ represents the Gibbs free energy of water. At 293 K and 0.1 MPa, ∆G_Water–water_ is −1500 cal/mol [33]. Under ambient conditions, the average volume of a water molecule is approximately 3 × 10^−29^ m^3^. Assuming it is a sphere, r_H2O_ is 1.9 Å.

In thermodynamics, the stability of a system is inversely related to its hydration free energy: the lower the hydration free energy, the more stable the system. The hydration free energy comprises the sum of ΔG_Water–water_ and ΔG_Solute–water_. It may be dominated by either ΔG_Water–water_ or ΔG_Solute–water_, depending on the size of the dissolved solute. Thus, a structural transition is anticipated to occur when ΔG_Water–water_ equals ΔG_Solute–water_.

(4) $${\Delta G}_{\mathrm{Water}–\mathrm{water}}={\Delta G}_{\mathrm{Solute}–\mathrm{water}}\;\;(\mathrm{Rc}=\frac{8\cdot {\Delta G}_{\mathrm{DDAA}}\cdot r_{H_{2}O}}{{\Delta G}_{\mathrm{Water}–\mathrm{water}}})$$

where Rc represents the critical radius of the solute [16]. At 293 K and 0.1 MPa, Rc is 6.5 Å for a sphere hydrophobic solute (Figure 1). As the size of the solute increases, it undergoes initial and hydrophobic solvation processes. Furthermore, ΔG_Solute–water_ is proportional to the ratio of the surface area to the volume of solute (1/R). Consequently, various solute dissolution behaviors in water may be expected during the initial and hydrophobic solvation processes.

In the initial solvation process, where ∆G_Solute–water_ is less than ∆G_Water–water_, hydration free energy is primarily governed by ∆G_Solute–water_ [16,17]. To achieve thermodynamic stability, maximizing the absolute value of ∆G_Solute–water_ is crucial. Consequently, dissolved solutes tend to disperse in aqueous solutions, with water molecules expected to exist between the solutes. However, during the hydrophobic solvation process, the Gibbs free energy of interfacial water exceeds that of bulk water (∆G_Solute–water_ > ∆G_Water–water_). To achieve more thermodynamic stability, maximizing the magnitude of ΔG_Water–water_ becomes crucial. This is accompanied by the minimization of the Gibbs free energy of interfacial water (|ΔG_Solute–water_|). Consequently, dissolved solutes tend to aggregate in solutions to minimize their surface area-to-volume ratio [16,17].

Due to hydrophobic interactions, the solutes are attracted and aggregated in aqueous solutions to maximize the hydrogen bonds of water. As solute surfaces come into contact, the available surface area for interfacial water inevitably decreases. Consequently, as solutes accumulate in water, the Gibbs free energy associated with interfacial water can be expressed as follows:

(5) $${\Delta G}_{\mathrm{Interfacial}\;\;\mathrm{water}}=\gamma \cdot {\Delta G}_{\mathrm{Solute}–\mathrm{water}}$$

in which γ represents the geometric factor [17]. It is introduced to reflect the changes in solute surfaces available for interfacial water as solutes aggregate in solutions. In fact, during the time in which solutes associate in water, changes in solute volume may also occur. Consequently, γ can be given as follows:

(6) γ=(Surface areaVolume)Aggregate(Surface areaVolume)Non–aggregate=f(1rSeparation)

in which r_Separation_ denotes the distance between the solutes. When solute surfaces come into contact, the corresponding distance between them is termed the hydrophobic radius (R_H_) [17]. With reference to R_H_ (or γ), the hydrophobic solvation process can be reasonably divided into H1w and H2s hydrophobic solvation processes.

In the H1w hydrophobic process, the separation between solutes exceeds R_H_ (>R_H_), or γ equals 1 [11], and no molecular surface aggregation is anticipated. As the distance between solutes decreases, water molecules within the inter-solute region are expelled into bulk water. Consequently, energy barriers may arise in the H1w process, likely due to the expulsion of water molecules. As a result, dissolved solutes are expected to approach each other in the direction with the lowest energy barrier, where fewer water molecules are expelled [34]. However, in the H2s hydrophobic process, the surfaces of dissolved solutes come into contact, resulting in γ being less than 1 (γ < 1). The aggregation of solute surfaces inevitably reduces the available surface area for interfacial water. To achieve greater thermodynamic stability, the solutes may aggregate to minimize their surface area-to-volume ratio. Thus, in the H2s solvation process, the directional nature of hydrophobic interactions is also expected. Consequently, different directional tendencies are found in the H1w and H2s processes [34].

During the aggregation of solutes in water, a phenomenon akin to the liquid–gas phase transition, known as the dewetting transition, may be observed [35,36]. To date, dewetting has been observed in numerous theoretical simulations involving nanotubes, plates in water [37,38], water–protein interfaces [39], and collapsing polymers [40]. From our recent study [17,18], dewetting may be closely associated with H2s hydrophobic processes, wherein a single water layer between solutes is expelled, leading to solutes making contact in solutions.

As solutes associate in solutions, interfacial water molecules within the region between solutes may be expelled into bulk water, which may be closely associated with hydrophobic interactions. Therefore, the strength of hydrophobic interactions may be dependent on the number of water molecules transitioning from interfacial to bulk water, which can be expressed as follows:

(7) $$\begin{array}{ll} {\Delta G}_{H} & =\gamma \cdot \frac{8\cdot {\Delta G}_{\mathrm{DDAA}}\cdot r_{H2O}}{R_{\mathrm{Solute}–\mathrm{solute}}}–\sum_{i=1}^{n}\frac{8\cdot {\Delta G}_{\mathrm{DDAA}}\cdot r_{H2O}}{R_{i}}\\ & \propto n_{\mathrm{Interfaical}\to \mathrm{bulk}\mathrm{water}}\cdot \left({\Delta G}_{\mathrm{Bulk}\mathrm{water}}–{\Delta G}_{\mathrm{Interfacial}\mathrm{water}}\right)\\ & =n_{\mathrm{Interfacial}\to \mathrm{bulk}\mathrm{water}}\cdot {\Delta G}_{\mathrm{DDAA}} \end{array}$$

Here, the first (second) term represents the Gibbs energy of interfacial water after (before) solutes are aggregated in aqueous solutions. Additionally, n_Interfacial→bulk water_ represents the number of water molecules transitioning from interfacial to bulk water during the association of solutes in solutions.

Owing to hydrophobic interactions, dissolved solutes tend to aggregate in aqueous solutions. Maximizing hydrophobic interactions may lead to the minimization of the surface area-to-volume ratio of solutes. To achieve greater thermodynamic stability, more interfacial water is expected to transition into bulk water as solutes aggregate in water. Because the solutes primarily affect the structure of interfacial water, the strength of hydrophobic interactions is closely linked to the geometric characteristics of the solute, such as its shape and size. For instance, when a sphere is associated with solutes having various surfaces, it tends to aggregate preferentially with convex surfaces compared to flat or concave surfaces.

When solutes are immersed in water, they must first approach each other before they can experience direct solute–solute interactions, such as van der Waals forces. Undoubtedly, hydrophobic interactions play a pivotal role as fundamental driving forces when solutes aggregate in solutions. Additionally, hydrophobic interactions may be related to the geometric characteristics of the solute, including its size and shape. Therefore, manipulating the geometric characteristics of solutes, such as their size and surface shape, can be applied to engineer “designed materials”.

Hydrophobic interactions are the most important nonspecific interactions and are regarded to play an important role in the self-assembly process of amphiphilic molecules in water [41,42,43,44]. This work may be applied to understand the concept of molecular packing parameters. The parameter was proposed by Israelachvili, Mitchell, and Ninham [2], which is defined as v_0_/aI_0_, where v_0_ and I_0_ are the volume and the length of the surfactant tail and a is the surface area of the hydrophobic core of the aggregate expressed per molecule in the aggregate. It is well known that the following connection exists between the molecular packing parameter and the aggregate shape: 0 ≤ v_0_/aI_0_ ≤ 1/3 for sphere, 1/3 ≤ v_0_/aI_0_ ≤ 1/2 for cylinder, and 1/2 ≤ v_0_/aI_0_ ≤ 1 for bilayer [3]. Thus, upon determining the molecular packing parameter, the shape and size of the equilibrium aggregate become readily discernible. This underscores the relationship between hydrophobic interactions and the geometric shape of interacting particles.

In the hydrophobic solvation process, the Gibbs energy of bulk water (ΔG_Water–water_) is lower than that of interfacial water (ΔG_Solute–water_), indicating a thermodynamically favorable state for solute aggregation in solutions. It seems that “attractive” forces exist between them. During the time in which the solutes are accumulated in solutions, a transition from interfacial to bulk water occurs. To maximize the hydrogen bonding of water, the solutes tend to aggregate to minimize their surface area-to-volume ratio. Therefore, hydrophobic interactions may be dependent on the geometric shape of the solute.

### 2.2. MD Simulations

In mathematics, the curvature of a solute surface can be roughly categorized as convex, flat, and concave. Moreover, the minimum ratio of surface area to volume is expected when the sphere aggregates with the concave surface of the solute. From the discussion on the dependence of hydrophobic interactions on the surface shape of solute, different hydrophobic interactions may be expected, as a sphere is associated with the various surfaces mentioned above. This variation may be related to the transformation of water molecules from interfacial to bulk water as the sphere is accumulated with the solute surface.

**Figure 2:**
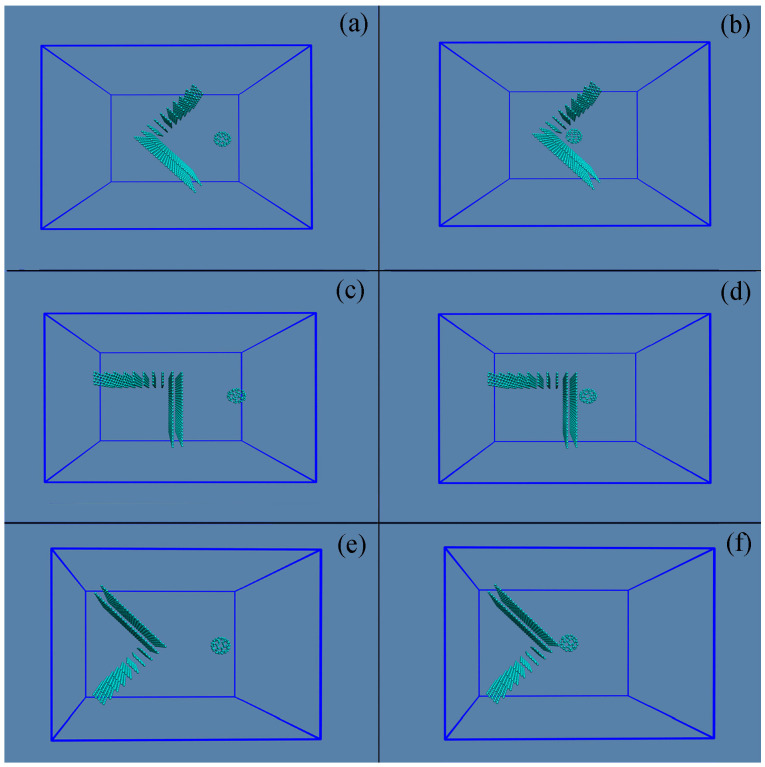
Simulated systems used to investigate the dependence of hydrophobic interaction on the geometric shape of the solute surface. A C_60_ is constrained to move to the concave (**a**,**b**), flat (**c**,**d**), and convex (**e**,**f**) surfaces of the target solute. Both initial and final configurations are shown.

In this work, to understand the dependence of hydrophobic interactions on the solute shape, a test solute is constrained to move to various surfaces of a target solute. The test solute is a C_60_ fullerene, and the target solute is graphite composed of different surfaces, such as concave, flat, and convex surfaces (Figure 2). In this study, during the time in which C_60_ fullerene is associated with various surfaces of the target solute, PMFs are calculated through umbrella sampling (US) [45,46,47] combined with the Weighted Histogram Analysis Method (WHAM) [48,49,50]. Based on the calculated PMFs, these may be utilized to understand the effects of surface shape on hydrophobic interactions.

**Figure 3:**
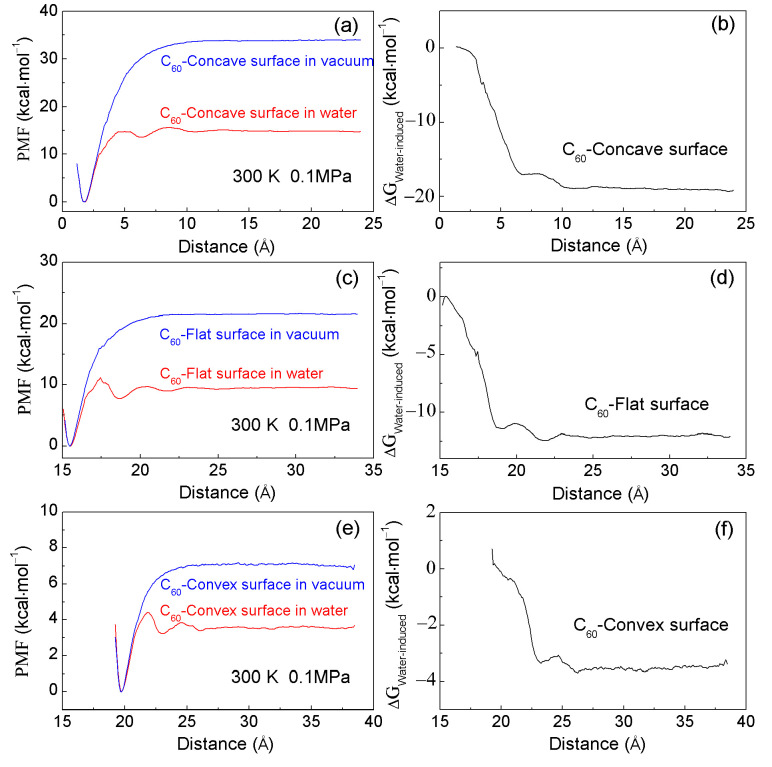
(**a**,**c**,**e**) The PMFs as the fullerene are associated with various surfaces of graphite in water and under vacuum at 300 K and 0.1 MPa. (**b**,**d**,**f**) The corresponding water-induced PMFs as solutes are aggregated in water.

According to the US calculations, the PMF profiles can be determined for the association of C_60_ fullerene with various surfaces of graphite in water (Figure 3). Upon decreasing the separation between C_60_ and graphite, three minima are observed in the calculated PMFs. The first minimum represents the contact minimum, with respective distances of 1.75 Å, 15.45 Å, and 19.75 Å for the C_60_-concave, C_60_-flat, and C_60_-convex surfaces. The second minimum corresponds to the solvent-separated PMF, indicating that only one water molecular layer can enter the space between the C_60_ fullerenes and graphite (Figure 3). These minima are located at 6.25 Å, 18.65 Å, and 23.05 Å, respectively. Additionally, a third minimum, positioned at 10.65 Å, 21.65 Å, and 23.05 Å, is also found in the calculated PMF curves, indicating the presence of a double water molecular layer between the test and target solutes (Figure 3). Furthermore, these are in accordance with other MD simulations [17,51,52,53,54] regarding the calculated PMFs of C_60_-C_60_ in water, and CH_4_-CH_4_ in water.

From Figure 3, energy barriers are observed between neighboring minima in the calculated PMFs. These barriers may arise from the expulsion of a single water layer within the confined volume as the solutes move closer together. This suggests that the water molecules between the solutes are progressively expelled into the bulk water, layer by layer, as the solutes approach each other. Consequently, the energy barrier is closely associated with the expulsion of water molecules during the association of solutes in solutions.

In addition, during the time in which the C_60_ is associated with various surfaces of graphite, obvious differences can be found in the calculated PMFs, especially at the first minimum, and the first energy barrier. Based on the calculated PMFs (Figure 3), the first minimum is ranked as follows: (first minimum)_C60-concave_ < (first minimum)_C60-flat_ < (first minimum)_C60-convex_. Additionally, the first energy barrier is listed as, (first energy barrier)_C60-concave_ > (first energy barrier)_C60-flat_ > (first energy barrier)_C60-convex_. Therefore, as C_60_ is associated with graphite, PMFs are dependent on the shape of solute surfaces.

To understand the water-induced contributions (ΔG_Water-induced_) as C_60_ is associated with different surfaces of graphite in water, the PMFs between solutes in a vacuum are also calculated using the US method (Figure 3). From the calculated PMFs, the water-induced contributions can be determined as follows:

(8) $${\Delta G}_{\mathrm{Water}-\mathrm{induced}}={\Delta G}_{\mathrm{Total}}-{\Delta G}_{\mathrm{Solute}\mathrm{in}\mathrm{vacuum}}$$

Based on the calculated water-induced contributions (ΔG_Water-induced_) (Figure 3), these can be utilized to investigate the dependence of hydrophobic interactions on the solute shape during their association in solutions.

According to the calculated water-induced contributions (ΔG_Water-induced_), in combination with our recent studies on hydrophobic interactions [16,17], “attractive” hydrophobic interactions may be expected between the fullerene and graphite. Additionally, different ΔG_Water-induced_ values can be found as the C_60_ is associated with various surfaces of graphite. Therefore, the water-induced PMFs are dependent on the geometric shape of the solute surface. From Figure 3, they may be listed as follows: (ΔG_Water-induced_)_C60-concave_ > (ΔG_Water-induced_)_C60-flat_ > (ΔG_Water-induced_)_C60-convex_. The strongest hydrophobic interactions are expected as C_60_ is aggregated with the concave surface of the target solute, which is also in accordance with the minimization of the surface area-to-volume ratio of solutes.

**Figure 4:**
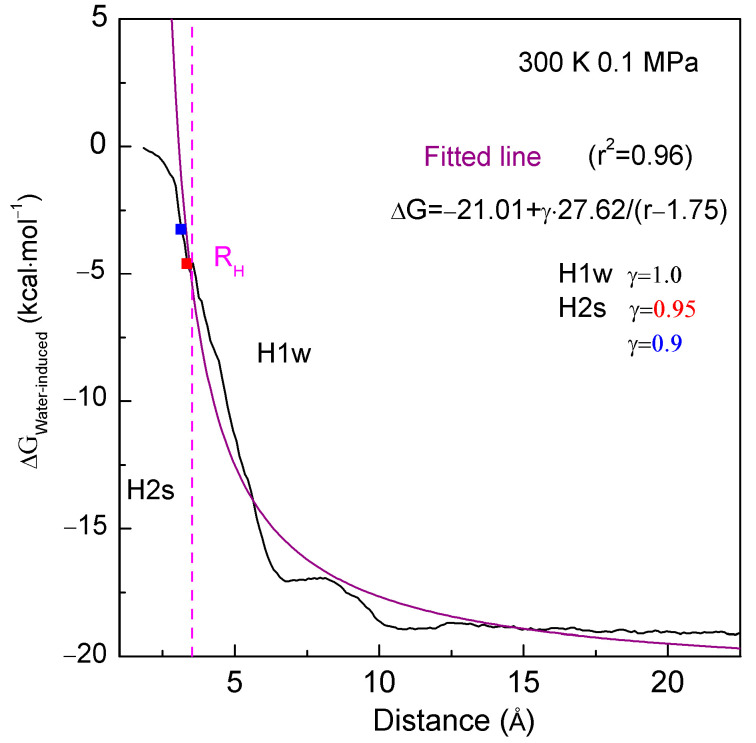
The water-induced PMFs as the C_60_ is aggregated with the concave surface of graphite at 300 K and 0.1 MPa. It is fitted as ΔG_Water−induced_ = a + b/(r − 1.75). In reference to R_H_ (hydrophobic radius), the hydrophobic interactions are divided into H1w and H2s hydrophobic processes. During the H1w process, γ = 1. During the H2s process, solute surfaces begin contact in water, and γ < 1.

From the above discussion, when C_60_ associates with graphite, the hydrophobic interactions may depend on the separation between them. During the time in which C_60_ associates with the concave surface of graphite, hydrophobic interactions can be fit as a function of the distance (r) between fullerene and graphite (Figure 4):

(9) $${\Delta G}_{\mathrm{Water}–\mathrm{induced}}=a+\gamma \cdot b/\left(r-r_{0}\right)$$

where r_0_ is the solute–solute distance at the first minimum of the PMFs when solutes are aggregated in a vacuum, and r − r_0_ means the separation between them. Additionally, the coefficients a and b are fitted to be −21.01 and 27.62, respectively.

In the works of Ball [55,56], water is recognized as an active participant in cell biology. It is widely acknowledged that water plays a crucial role in mediating hydrophobic interactions. During the hydrophobic solvation process, dissolved solutes are attracted and tend to aggregate within the water. As the distance between solute molecules decreases, water molecules within the region between them may be forced out into the bulk water. To understand the dependence of hydrophobic interactions on the geometric shape of the solute surface, it is essential to investigate the rearrangement of water molecules when fullerene associates with various surfaces of graphite in aqueous solutions.

As a solute becomes immersed in water, an interface emerges between the solute and water, inevitably influencing the structure of water. Recently, numerous experimental studies [57,58,59,60,61] have been conducted to investigate the effects of dissolved solutes on water structure. These studies suggest that the effects of ions on water are primarily confined to the first solvation shell. Indeed, this observation can also be elucidated through the dependence of OH vibrations on the hydrogen bonding network of water [23]. Consequently, the dissolved solute mainly affects the structure of interfacial water at the solute–water interface [23].

**Figure 5:**
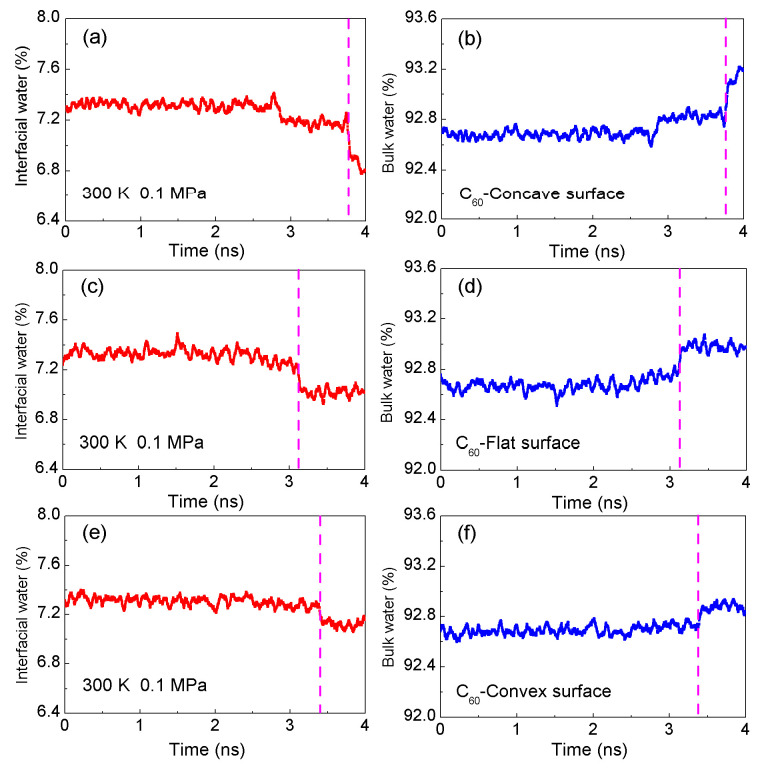
The changes in the interfacial and bulk water as C_60_ is associated with the concave (**a**,**b**), flat (**c**,**d**), and convex (**e**,**f**) surfaces of graphite in water at 300 K and 1 bar. The dashed line represents the corresponding time of R_H_.

From the MD simulations, decreasing the distance between the C_60_ and the solute surface leads to a decrease in the amount of interfacial water, but an increase in that of bulk water (Figure 5). Therefore, as solutes are aggregated in water, the interfacial water may be expected to change into bulk water. In other words, regarding the hydrophobic interactions, they may be closely related to the transition from interfacial to bulk water. In addition, the obvious changes from interfacial to bulk water, dewetting, is found as the separation between solutes, R_H_ (Figure 5). With a decreasing solute–solute distance, this is divided into H1w and H2s hydrophobic processes.

In the H1w hydrophobic process, water molecules may be found between solutes. When decreasing the amount of separation between solutes, the water molecules in the region between them may be expelled into bulk water. Therefore, this relates to the molecular reorganization between interfacial and bulk water during the H1w process. However, the solute surfaces begin to become a contact in the H2s process. In combination with our recent studies [17,18], dewetting may be closely related to H2s hydrophobic processes, in which a single water layer between solutes is expelled, and solutes come into contact in aqueous solutions.

Additionally, in our recent study [34], various directional natures are found in the H1w and H2s processes. In the H1w process, dissolved solutes are expected to approach each other in a specific direction characterized by the lowest energy barrier, resulting in fewer expelled water molecules. In the H2s process, to maximize the hydrogen bonding of water (or bulk water), the dissolved solutes are expected to aggregate in order to minimize the surface area-to-volume ratio. It is inferred that C_60_ tends to pass through the corners (or edges) of graphite and to be aggregated with the concave surface of graphite. This is supported by free MD simulations conducted during their accumulation in water (Supplementary Materials).

**Figure 6:**
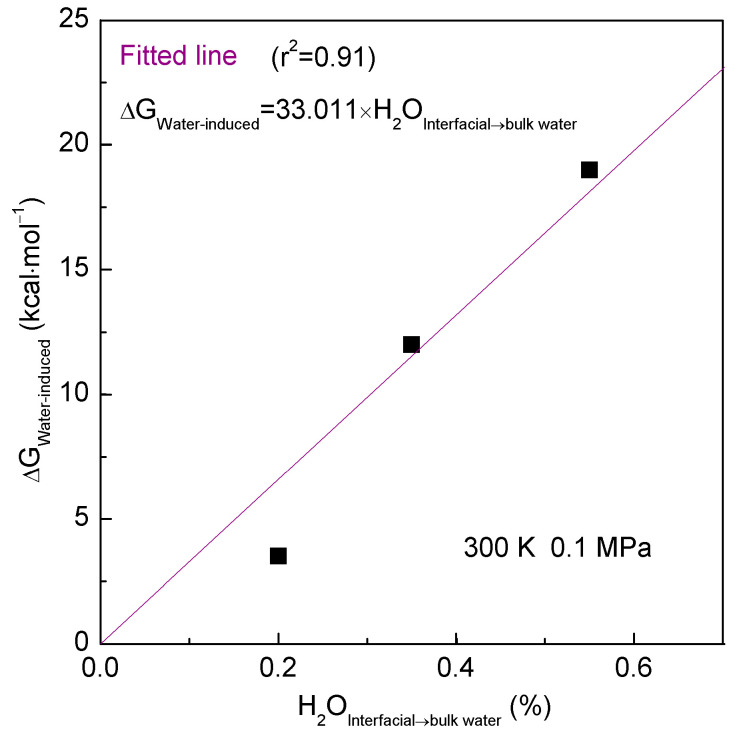
The relationship between water-induced PMFs and expelled water molecules from interfacial to bulk water as C_60_ is associated with various surfaces of graphite.

Various hydrophobic interactions may be found as C_60_ associates with different surfaces of graphite (Figure 4). Consequently, hydrophobic interactions exhibit dependency on the geometric shape of the solute surface. Additionally, based on MD simulations, as the sphere aggregates with various surfaces of graphite, the number of water molecules transformed from interfacial to bulk water (H_2_O_Interfacial→bulk water_) can be determined. It is evident that the ΔG_Water-induced_ is proportional to the H_2_O_Interfacial→bulk water_ (Figure 6). This is in agreement with the theoretical analysis of the effects of the geometric shape of the solute surface on hydrophobic interactions, as discussed above.

Due to hydrophobic interactions, dissolved solutes tend to be aggregated in aqueous environments, resulting in the transition from interfacial to bulk water. This transition highlights the relationship between hydrophobic interactions and the transformation of water molecules from interfacial to bulk water during solute aggregation. Because the solutes mainly affect the structure of interfacial water, the expulsion of water molecules during the transition from interfacial to bulk water may depend on the geometric shape of the solute surface. From the simulations, compared to flat and convex graphite, more water molecules are transformed from interfacial to bulk water when C_60_ is associated with the concave surface of graphite (Figure 5). Therefore, the strongest hydrophobic interactions are expected between the fullerene and the concave surface of graphite.

To maximize the hydrogen bonding of water (or bulk water), the dissolved solutes are expected to aggregate in order to minimize the surface area-to-volume ratio. This strategy corresponds to a specific orientation where more interfacial water molecules can be transformed into bulk water during the accumulation of solutes in aqueous solutions. In fact, the directional nature observed in the H2s process can be attributed to the maximization of hydrophobic interaction strength as solutes aggregate in aqueous solutions.

Due to the formation of hydrogen bonds between water molecules, water is generally regarded as an anomalous liquid. To date, various definitions of hydrogen bonds have been proposed. In this work, the geometrical definition of hydrogen bonding is employed to determine whether a hydrogen bond is formed or not [62]. Specifically, a hydrogen bond is considered to exist between two neighboring water molecules when the oxygen–oxygen distance (R_OO_) is less than 3.5 Å, and the ∠OOH angle between the two water molecules is less than 30°. In this study, the hydrogen bonding of water is calculated using the Visual Molecular Dynamics program [63].

**Figure 7:**
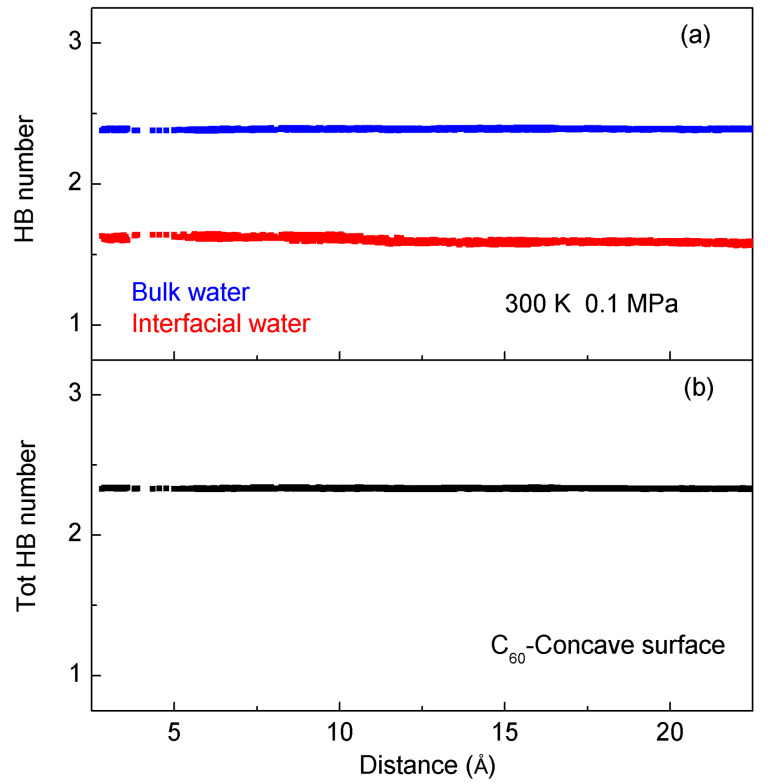
Hydrogen bonding number per water molecule in interfacial and bulk water (**a**) and total water (**b**) as the fullerene is aggregated with the concave surface of graphite at 300 K and 0.1 MPa.

From the MD simulations, the average number of hydrogen bonds per water molecule (n_HB_) can be determined during the association of C_60_ with the concave surface of the target solute (Figure 7). Compared to bulk water, a lower number of hydrogen bonds are observed in interfacial water. This is due to the truncation of hydrogen bonds at the solute–water interface. Regarding the origin of hydrophobic interactions, it is ascribed to the hydrogen bonds of bulk water being stronger than those of interfacial water. To maximize the hydrogen bonding of water, the dissolved solutes tend to aggregate in solutions. From a thermodynamic perspective, this aggregation process can be reasonably considered as an enthalpic process.

**Figure 8:**
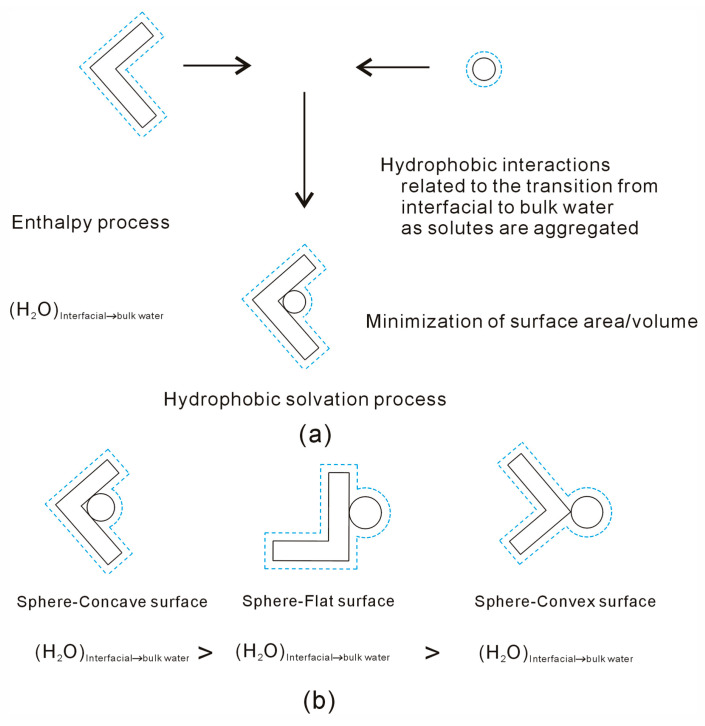
(**a**) Owing to hydrophobic interactions, the solutes are aggregated to minimize their surface area-to-volume ratio. (**b**) Hydrophobic interactions, related to the water molecules transformed from interfacial to bulk water, may be dependent on the solute shape.

Due to hydrophobic interactions, the solutes are attracted to form the aggregate in water (Figure 8). While the solutes are aggregated in water, the interfacial water molecules between solutes may be expelled into bulk water, which may be related to the strength of hydrophobic interactions. To maximize the hydrogen bonding of water, the solutes may be aggregated to minimize their surface area-to-volume ratio. The dissolved solutes mainly affect the hydrogen bonding of interfacial water. Therefore, hydrophobic interactions are dependent on the geometric shape of the solute (Figure 8).

## 3. Method

### 3.1. Simulated Systems

In this study, MD simulations were performed utilizing the NAMD program [64]. The test solute was C_60_ fullerene and the target solute was graphite composed of different surfaces, such as concave, flat, and convex surfaces. To investigate the dependence of hydrophobic interaction on solute shape, the sphere is constrained to move to the various surfaces of the target solute. The simulations were conducted under the isobaric–isothermal ensemble (NPT). The temperature was maintained at 300 K using moderately damped Langevin dynamics. A pressure of 1 atm was achieved using a Langevin piston. The initial dimensions of the simulated box were 60 Å × 60 Å × 90 Å for the system, with periodic boundary conditions applied in all three directions of Cartesian space.

During the simulations, the empirical CHARMM force field [65] was used to characterize interatomic interactions. The three-point intermolecular potential (TIP3P) model, the default water model in NAMD, represented the water molecules. Non-bonded van der Waals interactions were truncated to zero between 10 and 12 Å. Long-range electrostatic interactions were accounted for using the particle mesh Ewald (PME) algorithm. Additionally, the equations of motion were integrated with a time step of 2 fs.

### 3.2. PMF Calculations

The calculation of the PMFs as the C_60_ is associated with various surfaces of the graphite is achieved through US [45,46,47] combined with the WHAM [48,49,50]. In this work, the graphite was fixed, and C_60_ fullerene was constrained to move to the different surfaces of the graphite. The reaction coordinate is the separation between them. In US calculations, the distance between solutes may be divided into several windows, and the width of each window is about 1.0 Å. In addition, the WHAM was carried out using Grossfield [66] software (version 2.0.10) with a tolerance of 10^−7^.

## 4. Conclusions

Based on our recent structural studies on water and the air–water interface, this research is dedicated to exploring the relationship between hydrophobic interactions and the shape of the solute surface. From this work, the following conclusions can be drawn:

(1) Solutes primarily impact the hydrogen bonds of interfacial water, which may be weaker than those of bulk water. Due to hydrophobic interactions, the solutes may be attracted and tend to be aggregated to maximize the hydrogen bonding of water. As solutes associate in water, the strength of hydrophobic interactions may be closely related to the water molecules transformed from interfacial to bulk water.
(2) It is inferred that hydrophobic interactions are influenced by the geometric shape of the solute. This dependence on the shape of the solute surface aligns with the directional nature of hydrophobic processes, suggesting that solutes dissolve in a manner that minimizes their surface area-to-volume ratio.
(3) Hydrophobic interactions exhibit a correlation with the geometric shape of dissolved solutes. This understanding can be applied to interpret the molecular packing parameter commonly utilized in studying the self-assembly behavior of amphiphilic molecules in aqueous solutions.

## Data Availability

Data are contained within the article and Supplementary Materials.

## Supplementary Materials

The following supporting information can be downloaded at the following location: https://www.mdpi.com/article/10.3390/molecules29112601/s1, MD simulations are conducted to study the pathway during a C_60_ is associated with the graphite with different surfaces such as convex, flat, and concave. The simulated trajectory is provided in Supplementary Data.
