# Supplementary material for: The Dependence of Hydrophobic Interactions on the Shape of Solute Surface
Source: Molecules. 2024 Jun 1;29(11):2601. doi: 10.3390/molecules29112601 (PMC11173737; doi:10.3390/molecules29112601)
Supplement: Supplementary file 1 [file molecules-29-02601-s001.zip › Supplementary.pdf]

Supplementary Materials

The Dependence of Hydrophobic Interactions on the Shape of Solute Surface

Yu-Zhen Liu, Yan-Nan Chen and Qiang Sun

Key Laboratory of Orogenic Belts and Crustal Evolution, Ministry of Education,

The School of Earth and Space Sciences, Peking University, Beijing 100871, China; liuyuzhen@stu.pku.edu.cn (Y.L.); 2301210129@stu.pku.edu.cn (Y.C.);

* Correspondence: QiangSun@pku.edu.cn

Owing to hydrophobic interactions, the solutes tend to be aggregated in solutions. The solutes mainly affect the structure of interfacial water. Additionally, hydrophobic interactions are related to the transition of water molecules from the interfacial region to the bulk water. Therefore, hydrophobic interactions may be dependent on the shape of the solute surface. To enhance the strength of hydrophobic interactions, solutes are expected to be aggregated to minimize their surface area-to-volume ratio. To investigate the effects of solute shape on hydrophobic interactions, MD simulations are conducted to study the pathway during a C_{60} is associated with the graphite with different surfaces such as convex, flat, and concave. It is found that the fullerene tends to be associated with the edge of graphite, and become aggregated with the concave surface of graphite. In the trajectories, only the interfacial water layers are shown.

MD simulations were conducted using NAMD 2.14 package. The simulations were carried out in the NVT ensemble. The simulated temperature was kept at 300 K. During the simulations, the empirical CHARMM force field was utilized to describe interatomic interactions. The water molecules were simulated using the intermolecular three point potential (TIP3P) water model. For each MD simulation, the simulated time was 12 ns, and the time step was 2 fs. The simulated results are analyzed through VMD package.
